# A Delayed Presentation of Salt-Wasting Congenital Adrenal Hyperplasia Due to 3β-Hydroxysteroid Dehydrogenase Deficiency in an Infant: A Case Report

**DOI:** 10.7759/cureus.108805

**Published:** 2026-05-13

**Authors:** Bayan Manaf Ghareeb, Fatema Naser Shakeeb, Maryam Busehail, Maha Alarrayedh

**Affiliations:** 1 Medicine, Royal College of Surgeons in Ireland, Busaiteen, BHR; 2 Genetics, Salmaniya Medical Complex, Manama, BHR; 3 Pediatric Endocrinology, Salmaniya Medical Complex, Manama, BHR

**Keywords:** 3β-hydroxysteroid dehydrogenase deficiency, adrenal crisis, ambiguous genitalia, congenital adrenal hyperplasia, delayed presentation, salt-wasting cah

## Abstract

Congenital adrenal hyperplasia (CAH) is a group of inherited disorders affecting adrenal steroid synthesis and can lead to adrenal insufficiency, salt-wasting crises, and disorders of sex development. Most cases are identified in the neonatal period; however, rarer enzymatic defects may present later, making diagnosis more challenging. We report a case of classical salt-wasting CAH most consistent with 3β-hydroxysteroid dehydrogenase type 2 (3β-HSD2) deficiency in a two-month-old infant who presented with sudden cardiac arrest due to adrenal crisis. The infant had been clinically well since birth, with no early features suggestive of adrenal insufficiency. Prompt recognition and initiation of glucocorticoid and mineralocorticoid therapy led to rapid clinical stabilization. This case highlights the marked variability in the clinical presentation of classical 3β-HSD2 deficiency and emphasizes that severe, life-threatening adrenal crises may occur beyond the neonatal period, even in the absence of early warning signs.

## Introduction

Congenital adrenal hyperplasia (CAH) comprises a group of autosomal recessive disorders caused by defects in enzymes involved in adrenal steroid biosynthesis. These defects result in impaired cortisol production, with variable effects on aldosterone and sex steroid synthesis. Although 21-hydroxylase deficiency accounts for the majority of CAH cases, less common enzymatic defects present a wider range of clinical features and may be more difficult to recognize [1].

Deficiency of 3β-hydroxysteroid dehydrogenase type 2 (3β-HSD2) is a rare form of CAH that disrupts the conversion of Δ5 to Δ4 steroids, leading to deficiencies of cortisol, aldosterone, and testosterone [2]. Classical disease typically presents in the neonatal period with salt-wasting crisis and ambiguous genitalia [3].

We report a case of classical salt-wasting CAH most consistent with 3β-HSD2 deficiency presenting at two months of age with cardiac arrest secondary to adrenal crisis. This unusually late and severe presentation highlights the need for ongoing clinical vigilance for adrenal insufficiency in young infants, even beyond the neonatal period and in the absence of obvious early manifestations.

## Case presentation

A two-month-old infant, initially assigned female at birth, was referred to a tertiary care center following a life-threatening salt-wasting crisis suspected to be due to CAH.

The infant was born at term via spontaneous vaginal delivery after an uncomplicated pregnancy. There were no antenatal or postnatal complications, and neonatal intensive care was not required. She was discharged on the second postnatal day as a healthy newborn. Feeding, growth, and early developmental milestones were reported as normal, and immunizations were up to date. The parents were first-degree cousins, with no family history of neonatal deaths, metabolic conditions, or known endocrine disease.

The patient initially developed recurrent vomiting for one week, followed by hypoactivity and poor feeding over the subsequent five days. During the initial medical evaluation, she suffered a cardiorespiratory arrest with a documented heart rate <60 beats per minute. Return of spontaneous circulation was achieved after five cycles of cardiopulmonary resuscitation, after which she was transferred for further management.

On arrival, the infant was critically ill, pale, and lethargic, with gasping respirations and marked chest retractions. She had a one-week history of vomiting, one day of markedly reduced oral intake, and acute respiratory distress associated with noisy breathing. There was no history of fever, cough, abnormal movements, trauma, or sick contacts. Oxygen saturation improved following intubation. She was tachycardic, with weak peripheral pulses, prolonged capillary refill time, and initially unrecordable blood pressure. Random blood glucose was low. Neurological examination revealed lethargy and hypotonia, with no dysmorphic features or neurocutaneous stigmata.

Genital examination revealed ambiguous genitalia with clitoromegaly, hyperpigmented labioscrotal folds, a single perineal opening, and bilaterally palpable gonads within the inguinal canals.

Initial arterial blood gas analysis demonstrated metabolic acidosis with significant hyponatremia and hyperkalemia (Table 1).

**Table 1:** Initial laboratory findings on presentation

Test	Value	Reference range	Interpretation
Sodium	108 mmol/L	135-145 mmol/L	Severe hyponatremia (salt-wasting crisis)
Potassium	9.7 mmol/L	3.5-5.5 mmol/L	Severe hyperkalemia (salt-wasting crisis)
pH	7.13	7.35-7.45	Metabolic acidosis
Bicarbonate	13.9 mmol/L	22-28 mmol/L	Metabolic acidosis
Blood glucose	3 mmol/L	3.9-6.1 mmol/L	Hypoglycemia
Blood pressure	62/36 mmHg	70-90/40-60 mmHg (age)	Consistent with shock/hypotension

An adrenal crisis secondary to salt-wasting CAH was strongly suspected, with sepsis considered an important differential diagnosis. Endocrine evaluation supported a diagnosis most consistent with 3β-HSD2 deficiency (Table 2). Given the emergent clinical presentation, corticosteroids were administered prior to obtaining baseline cortisol levels.

**Table 2:** Hormonal tests *Cortisol level reflects exogenous hydrocortisone administration

Test	Value	Reference range	Interpretation
Adrenocorticotropic hormone	171 pg/mL	7-63 pg/mL	Elevated; supports adrenal insufficiency, 3-beta-hydroxysteroid dehydrogenase deficiency
17-hydroxyprogesterone	30 ng/dL	30-90 ng/dL	Low-normal levels; argues against classic 21-hydroxylase congenital adrenal hyperplasia
Testosterone	4.85 ng/dL	3-8 ng/dL	Within reference; supports undervirilization
Androstenedione	0.5 ng/dL	0.2-3.5 ng/dL	Low-normal; consistent with 3-beta-hydroxysteroid dehydrogenase deficiency
Cortisol (post-steroid)*	1940 nmol/L	200-700 nmol/L	Elevated due to exogenous hydrocortisone

**Figure 1:**
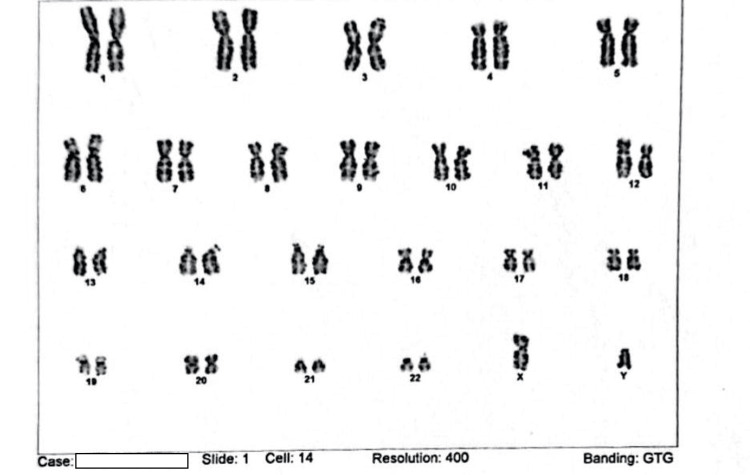
G-banded karyotype at 400-band resolution showing a normal male chromosomal complement (46,XY)

Tandem mass spectrometry, extended steroid profiling, karyotyping, and genetic testing were initiated as part of the diagnostic workup (Table 3, Figure 1).

**Table 3:** Molecular genetic findings

Gene (transcript)	Location	Variant	Zygosity	Disease (OMIM)	Inheritance	Classification
HSD3B2(+)(ENST00000369416.4)	Exon 4	c.745C>T (p.Arg249Ter)	Homozygous	Adrenal hyperplasia, congenital, due to 3-beta-hydoxysteroid dehydrogenase 2 deficiency (OMIM#201810)	Autosomal recessive	Pathogenic (PVS1, PM2, PP5)

Abdominal ultrasonography demonstrated normally sized kidneys with increased echogenicity. The right adrenal gland appeared normal, while the left adrenal gland was bulky. Bilateral inguinal testes were identified, with a small amount of fluid surrounding the right gonad. Pediatric surgical consultation advised deferring surgical intervention until at least one year of age.

Management included supplemental oxygen, intravenous dextrose and isotonic saline boluses, and treatment of hyperkalemia according to protocol. Broad-spectrum intravenous antibiotics were initiated following sepsis evaluation. A stat dose of intravenous hydrocortisone resulted in rapid improvement in hemodynamic status and neurological responsiveness. The patient was admitted to the pediatric intensive care unit, where stress-dose hydrocortisone and deficit-based intravenous fluids were continued with close monitoring of electrolytes, blood gases, glucose levels, vital signs, and urine output. A packed red blood cell transfusion was administered for anemia. Enteral feeding was gradually introduced and advanced as tolerated. Hydrocortisone was later transitioned to oral therapy, with the addition of fludrocortisone and oral sodium supplementation.

At discharge, the infant was clinically stable and feeding well, with normalization of serum electrolytes (Table 4).

**Table 4:** Selected follow-up laboratory findings upon discharge

Parameter	Reference range	Value	Interpretation
Sodium	135-145 mmol/L	134-138 mmol/L	Improved from severe hyponatremia (108 mmol/L)
Potassium	3.5-5.5 mmol/L	5 mmol/L	Decreased from severe hyperkalemia (9.7 mmol/L)
Bicarbonate	22-28 mmol/L	24 mmol/L	Corrected metabolic acidosis (initial 13.9 mmol/L)
Glucose	3.9-6.1 mmol/L	4 mmol/L	Corrected from initial hypoglycemia (3 mmol/L)

She was discharged on oral hydrocortisone (15 mg/m²/day in divided doses), fludrocortisone 100 µg once daily, and oral sodium chloride supplementation at 2 mmol/kg/day. Caregivers were counseled on medication adherence, stress-dose steroid administration, and recognition of symptoms of adrenal insufficiency.

At outpatient follow-up, the infant remained clinically well with appropriate growth. Medication adherence was good. Electrolytes remained stable. Physical examination revealed persistent ambiguous genitalia with a phallic structure measuring approximately 2 cm; only the right testis was palpable, a single perineal opening, and resolving genital hyperpigmentation. No hyperpigmentation of the lips or nail beds was noted, and the remainder of the systemic examination was unremarkable.

## Discussion

CAH encompasses a group of autosomal recessive disorders of steroidogenesis, most commonly caused by 21-hydroxylase deficiency, which accounts for approximately 90-99% of cases [1]. In contrast, 3β-HSD2 deficiency is an exceptionally rare form of CAH, accounting for less than 1% of cases, with fewer than 200 cases reported in the global literature and limited data on long-term outcomes [2]. Differentiating between these subtypes is clinically important due to differences in hormonal profiles and phenotypic presentation. Classic 21-hydroxylase deficiency is typically characterized by markedly elevated 17-hydroxyprogesterone levels and virilization of genetic females, whereas 3β-HSD2 deficiency results in impaired synthesis of glucocorticoids, mineralocorticoids, and sex steroids, leading to salt-wasting in both sexes and undervirilization in genetic males [4]. In this patient, the presence of severe salt wasting, ambiguous genitalia with undervirilization, and a non-elevated 17-hydroxyprogesterone level strongly favored 3β-HSD2 deficiency over classic 21-hydroxylase deficiency [5].

The condition is generally classified into classical and nonclassical forms. Classical disease, caused by pathogenic variants in the HSD3B2 gene, is associated with varying degrees of salt wasting and disordered sexual development, while nonclassical forms tend to present later with milder features such as premature pubarche or hyperandrogenism [4]. The study by Johannsen et al. [6] reported that in most cases, the classical salt-wasting form of 3β-HSD2 deficiency is identified in the neonatal period or early infancy, when life-threatening electrolyte disturbances prompt urgent evaluation, often in the setting of ambiguous genitalia, particularly in 46,XY infants.

Despite the expectation of early diagnosis, delayed presentation of classical 3β-HSD2 deficiency has been described, most often in patients with partial residual enzyme activity or subtle genital findings that do not initially raise concern [7,8]. In the present case, the infant remained clinically well for the first eight weeks of life before presenting abruptly with cardiac arrest due to adrenal crisis. This unusually late and severe presentation highlights the broad clinical variability of classical 3β-HSD2 deficiency. The case report by Özdemir et al. [8] and the clinical review by Al Alawi et al. [9] described delayed presentations of 3β-HSD2 deficiency in both infancy and adulthood, highlighting variability in age at diagnosis and disease severity; however, neither reported cardiac arrest as the initial manifestation during infancy. Similarly, the case reported by Saira Yousaf et al. [10] demonstrated prolonged misclassification as 21-hydroxylase deficiency until adulthood, despite biochemical features that were inconsistent with this diagnosis. In the case reported by Yousaf et al., persistently low 17-hydroxyprogesterone levels in the setting of elevated adrenocorticotropic hormone (ACTH) prompted further evaluation, ultimately leading to the diagnosis of 3β-HSD2 deficiency. Together with our findings, these reports underscore how rare CAH subtypes may remain unrecognized for decades when early diagnoses are not genetically confirmed, emphasizing the need for diagnostic reassessment when clinical and hormonal features are discrepant.

In our patient, the delayed but catastrophic presentation was most likely the result of progressive mineralocorticoid deficiency, leading to gradual sodium loss, worsening hyponatremia, hyperkalemia, and ultimately cardiorespiratory collapse secondary to severe electrolyte imbalance. Compared with other delayed presentations reported in the literature, the degree of electrolyte disturbance in this case was particularly severe, underscoring how unrecognized or partially compensated 3β-HSD2 deficiency can culminate in sudden, life-threatening collapse.

## Conclusions

This case demonstrates that classical salt-wasting CAH due to 3β-HSD2 deficiency may present beyond the neonatal period with sudden, life-threatening deterioration. A normal 17-hydroxyprogesterone level does not exclude CAH in rare enzymatic defects. Early recognition of adrenal insufficiency and prompt administration of hydrocortisone were critical to rapid clinical recovery. Clinicians should consider CAH in infants presenting with vomiting, shock, hypoglycemia, and electrolyte disturbances, as timely treatment can be lifesaving.
